# Effects of *Bunium persicum* essential oil on the reduction of spore germination, growth, and expression of *FUM1* and *FUM14* genes in *Fusarium verticillioides* isolates

**DOI:** 10.18502/CMM.7.2.7033

**Published:** 2021-06

**Authors:** Asad Balal, Aghil Sharifzadeh, Hojjatollah Shokri, Ali Reza Khosravi

**Affiliations:** 1 Mycology Research Center, Faculty of Veterinary Medicine, University of Tehran, Tehran, Iran; 2 Department of Pathobiology, Faculty of Veterinary Medicine, Amol University of Special Modern Technologies, Amol, Iran

**Keywords:** Antifungal activity, *Bunium persicum*, *FUM1* and *FUM14* genes, *Fusarium verticillioides*, Real time-PCR

## Abstract

**Background and Purpose:**

Black Cumin of Kerman (*Bunium persicum*) is an Iranian plant that is commonly used as an antispasmodic, carminative, and antimicrobial substance. The present study aimed to assess different components of the essence of *B. persicum* and its effect on antifungal activity, spore germination inhibition, and expressions of *FUM1* and *FUM14* genes in *Fusarium verticillioides* strains.

**Materials and Methods:**

The essence was extracted by hydrodistillation and analyzed through gas chromatography-mass spectroscopy. A broth microdilution method was used for the determination of the minimum inhibitory concentration (MIC). In addition, the expression of *FUM1* and *FUM14* genes of toxigenic *F. verticillioides* was assessed by using the real-time polymerase chain reaction (RT-PCR) technique.

**Results:**

Based on the findings, most of the essence consisted of γ-terpinene (15.56%), propanal, and 2-methyl-3-phenyl (14.18%). The oil showed a good antifungal activity (mean MIC value: 2556.8 μg/ml) as well as the inhibition of spore germination and mycelial growth (*P*<0.05). The RT-PCR demonstrated that the expression levels of *FUM1* and *FUM14* of *B. persicum*-treated *F. verticillioides* were 0.43 and 0.53 folds lower than the control samples, respectively.

**Conclusion:**

These findings revealed that the essential oil of *B. persicum* has different components responsible for the inhibition of mycelial growth and spore germination of *F. verticillioides* as well as reduction of expressions of *FUM1* and *FUM14* genes involving fumonisin production.

## Introduction

*Fusarium verticillioides* is considered one of the most frequent *Fusarium* species that contaminate various crops, in particular maize [ 1 , 2 ]. Previous studies have demonstrated the high variability of molecular and genetic properties of *F. verticillioides*, resulting in variation in its pathogenicity [ 3 ]. Mycotoxins produced by *F. verticillioides* (especially fumonisins) may result in various diseases, such as the appearance of black points on crops, leukoencephalomalacia, and immune dysfunction in animals and mycotoxicosis in humans [ 3 , 4 ]. Therefore, *F. verticillioides* strains are one of the most important plant-infecting fungi with toxigenic and pathogenic potential on animal and human health.

Plants have different bioactive sources of a wide variety, such as tannins, terpenoids, saponins, alkaloids, and flavonoids [ 5 ]. Hence, the search for antifungal usage of plant-derived compounds has accelerated in recent years due to their importance in drug discovery [ 6 ]. *Bunium persicum* (Boiss.) B. Fedtsch (Black Cumin of Kerman) is a member of the Apiaceae family and is an herbal plant that naturally grows in arid regions of Iran [ 7 ]. Antimicrobial [ 8 ] and antifungal [ 9 ] effects of this plant have been proved in previous studies. 

In a study conducted by Sekine et al. [ 9 ], *B. persicum* showed potent antifungal activity against phytopathogenic fungi. Although different investigations demonstrated the growth inhibition of various fungi, there has been no report on the effect of *B. persicum* essence on morphological and genetic changes of *F. verticillioides*. Therefore, the current study aimed to investigate the efficacy of *B. persicum* essence in the reduction of spore germination, growth, and expression of *FUM1* and *FUM14* genes in *F. verticillioides* strains. 

## Materials and Methods

### Strains

In total, 20 strains of toxigenic *F. verticillioides* were used in this study. All strains were isolated from fumonisin-infected livestock and poultry feeds and validated by molecular methods at the Faculty of Veterinary Medicine, University of Tehran, Iran.

### Extraction and identification of Bunium persicum oil components

Extraction of essence from *B. persicum* seeds was carried out by hydrodistillation method using a Clevenger device (Biogenic, Brasilia, Brazil) [ 10 ]. After collecting and drying the essence over anhydrous sodium sulfate, it was stored in a sterilized vial at 4 °C until use. The extracted oil was analyzed using gas chromatography-mass spectroscopy (Agilent Technologies, Avondale, PA, USA). Briefly, the essence (1 µl) was injected into the capillary column and analyzed by MSD Chemstation Software (version E.02.02) (Agilent Technologies). In temperature programming, the temperature profiles were adjusted at 50 °C for 2 min, then the temperature increased from 25 °C min^-1^ to 100 °C and was kept there for 2 min, and after that, the temperature increased from 5 °C min^-1^ to 290 °C where it was kept for 5 min. Subsequently, helium was held at a constant flux of 1.0 ml min^-1^. After chromatographic separation, several peaks were seen and analyzed in our computer library. 

### Determination of minimum inhibitory and fungicidal concentrations of Bunium persicum essence

The *in vitro* antifungal assay was determined in a 96-well microtiter plate bioassay according to the instructions of the Clinical and Laboratory Standards Institute M38-A2 regarding the broth microdilution method in three replicates with slight modifications [ 11 ]. Fungal suspensions were prepared from fresh and mature (5-day-old) cultures grown on Potato dextrose agar (PDA) (Merck Co., Darmstadt, Germany) slants at 37 °C covered with 5 ml of RPMI 1640 medium supplemented with 0.05% Tween 80. 

After gentle vortexing, the suspensions were filtered and diluted in RPMI 1640, adjusting the concentration of 1×10^5^ conidia/ml. Aliquots of 100 µl of various concentrations (diluted in dimethyl sulfoxide 5%) of *B. persicum* (500, 1000, 1500, 2000, 2500, 2750, 3000, 3250, 3500, and 4000 µg/ml) were dispensed into 96-well plates. Subsequently, 100 µl of diluted conidial inoculum suspensions were added to each well of a plate. Microdilution plates were incubated at 35 °C and examined after 48 h for determination of the minimum inhibitory concentration (MIC). The MIC was considered the lowest concentration of *B. persicum* essence that was required for total growth inhibition of *F. verticillioides* isolates.

*The minimum fungicidal concentration (MFC) was defined* as the lowest essence level in which *no visible growth* occurred when *subcultured* onto Sabouraud dextrose agar (SDA) plates. It should be mentioned that the media were kept at 35 °C for 48 h. Control wells were prepared with RPMI 1640 media; positive control contained broth medium and *F. verticillioides* suspension while negative control contained broth medium and test compound. The MFC/MIC ratio was calculated to determine whether *B. persicum* essential oil has a fungistatic (MFC/MIC≥4) or fungicidal activity (MFC/MIC<4). 

### Effect of Bunium persicum on radial mycelial growth

Effect of *B. persicum* essence on the radial mycelial development was evaluated using the poisoned substrate method. For this purpose, a 5 mm diameter plug was taken from a 7-day-old *F. verticillioides* cultivated on PDA and put at the center of the SDA Petri dishes containing *B. persicum* oil at MIC_90_, ½ MIC_90_, and 2MIC_90_ and incubated at 30 °C. Plates without essence and with itraconazole (MIC: 8 µg/ml) were considered negative and positive controls, respectively. The diameters (mm) of the radial growth were recorded at different time points (1, 2, 4, 6, 8, 10, 12, and 14 days) during incubation [ 12 ].

### Relative fungal inhibition of Bunium persicum essence

Plates containing *B. persicum* oil at MIC_90_, ½ MIC_90_, and 2MIC_90_ concentrations along with untreated control plates were inoculated with a 5-mm agar disc of an actively growing *F. verticillioides* culture. It should be mentioned that three sets of assays were performed per treatment. Fungal growth (colony diameter) was measured after 7 days of incubation at 30 °C and relative fungal inhibition was calculated according to the following formula:

Relative fungal inhibition (%)=(C-T) x 100/C

C=colony diameter (mm) of the control 

T=colony diameter (mm) of the test plate

### Effect of Bunium persicum essence on spore germination

Effect of *B. persicum* oil on spore germination of *F. verticillioides* was analyzed using a method developed by Kalagatur et al. [ 13 ]. Aliquots (10 μl) of conidial suspension (1×10^6^ conidia/ml) were obtained from 10-day-old cultures, cultured onto SDA slides containing various concentrations of *B. persicum* (500, 1000, 1500. 2000, 2500, 2750, 3000, 3250, 3500, and 4000 µg/ml) and stored at 28 °C. After 24 h, each slide was stained using lactophenol-cotton blue and examined for spore germination. It must be noted that a control group without essence was used as well. In total, 200 spores were evaluated from each slide and spore germination percentage was calculated as follows: spore germination %=ST/SC×100. The SC and ST indicate the number of spores germinated in the control and test groups, respectively.

### Isolation of mRNA from Fusarium verticillioides strains treated with Bunium persicum and reverse transcription

*F. verticillioides* isolates were cultured on PDA with 2000 μg/ml of *B. persicum* oil. The mycelia were ground for 20 s at 1500 rpm several times with a Multi-Beads Shocker (Yasui Kikai, Osaka, Japan) in liquid nitrogen. It must be mentioned that three replicates for each treatment were performed. RNeasy Plant Mini Kit (Qiagen, Hilden, Germany) was used to extract total RNA. Subsequently, the quality of RNA extracts was assessed spectrophotometrically and the integrity of RNA was verified by agarose gel electrophoresis with 1.2% agarose gel and stored at -80 ºC. The contaminating genomic DNA was removed using RNase-free DNAse I (Qiagen). Reverse transcriptase Omniscript Reverse Transcription (Qiagen) was used to synthesize the first-strand cDNA. A final reaction mixture (20 µl) was composed using 2 µl of 10×RT-polymerase chain reaction (RT-PCR) buffer, 2 µg of total RNA, 1 µl (4U) of RNAse inhibitor, 2 µl of oligo d(T) 16 (10 µM), 2 µl of dNTPs (10 mM), 1 µl (4U) of Omniscript Reverse Transcriptase and RNase free water. The cDNA synthesis was carried out at 37 ºC for 60 min [ 6 ].

### Real-time polymerase chain reaction

Real-time PCR was used to analyze the efficacy of *B. persicum* essence on the expressions of *FUM1* and *FUM14* genes which are involved in fumonisins biosynthesis in the strains of *F. verticillioides*. *CALM* (calmodulin) was used as an endogenous reference gene to normalize the expression levels. The RT-PCR primer sequences used for this were: *FUM1-F* (5'-GAGCCGAGTCAGCAAGGATT-3') and *FUM1-R* (5′-AGGGTTCGTGAGCCAAGGA-3′) according to the *FUM1* (fragment size 90bp) gene sequence (NCBI GenBank Accession No. AF155773) as well as *FUM14-F* (5'-TAGGTCCAGGTCGAGATGCT-3') and *FUM14-R* (5'-GGAAGCCAAGAACCCAATCT-3') according to the *FUM14* (fragment size 99bp) gene sequence (NCBI GenBank Accession No. AF155773). *CALM-F* (5'-TCAGCCTCTCGGATCATCTC-3') and *CALM-R* (5'-TCAGCCTCTCGGATCATCTC-3') were used to amplify the *CALM* (fragment size 97 bp) transcript [ 14 ].

The PCR amplification was performed using Rotor Q (Qiagen Co., Hilden, Germany) system. The qRT-PCR thermal cycling conditions were 94 ºC for 5 min, 94 ºC for 30 sec, 60 ºC for 30 sec with a final extension at 72 ºC for 45 sec; in total, 40 cycles were performed. The final reaction mixture (25 µl), SYBER® Green PCR Master Mix (Applied Biosystems, USA), was composed of 1.5 μl of each primer (5 μM), 4.5 μl of sterile Milli-Q water, and 5 μl of template cDNA. Negative controls were subjected to all the experiments as well. The samples were run in triplicate in each experiment for all the tested genes. In this study, the analysis method was relative quantification according to ΔΔCt values.

### Statistical analysis

One-way ANOVA (Sigma Stat, version 3.5) was used to compare the effects on various *F. verticillioides* strains. It should be noted that a *p*-value of less than 0.05 was considered statistically significant.

## Results and Discussion

The limitations in using synthetic antifungals and the probability of the development of resistance to these fungicidal agents prompted us to search for natural antifungals with no side effects on human health. For this purpose, we used *B. persicum* essence for *F. verticillioides* strains. The gas chromatography-mass spectrometry results of *B. persicum* essence are summarized in Table 1. In total, 35 components were identified that represented 98.64% of the total oil. Moreover, γ-terpinene (15.56%), propanal, and 2-methyl-3-phenyl (14.18%) were found as its major components. Previous studies have reported different biological activities of *B. persicum* grown in Iran. Results of this study are in accordance with those of the investigations performed by Khaledi and Hassani [ 15 ], Sharafati Chaleshtori et al. [ 16 ], and Shahsavari et al. [ 17 ] on the *B. persicum* oil collected from Iran. Although the same basic components were present in all essence samples, some elements were found with different concentrations. Various findings demonstrate the influence of climatic and local factors on essence components [ 18 ] as well as the difference between the wild and domesticated species of *B. persicum* [ 19 ]. Therefore, the origins or genetic backgrounds of *B. persicum* could affect their *biological and pharmaceutical activities*.

**Table 1:** Chemical compositions of the *Bunium persicum* essence analyzed by gas chromatography-mass spectroscopy

Number	Components	Retention time (min)	Area (%)
1	α-phellandrene	3.211	0.60
2	Trans-β-Ocimene	3.367	3.53
3	Camphene	3.673	0.29
4	Sabinene	4.41	9.12
5	β-myrcene	4.482	0.86
6	α-terpinene	5.634	0.91
7	Cymene	5.972	6.50
8	δ-3-carene	6.055	0.68
9	γ-terpinene	7.31	15.56
10	Cis-sabinene hydrate	7.466	0.49
11	α-terpinolene	8.234	0.56
12	Terpineol, z-beta	8.592	0.39
13	Linalool L	8.815	0.95
14	Citronella	11.01	2.01
15	4-terpineol	11.876	2.55
16	β-fenchyl alcohol	12.468	1.52
17	m-diethyl benzene	13.916	0.24
18	Propanal, 2-methyl-3-phenyl	14.694	14.18
19	Geraniol	15.462	0.94
20	Z-citral	16.074	0.44
21	2-caren-10-al	16.401	3.67
22	Myrtenal	16.883	9.52
23	Carvacrol	17.516	0.23
24	P-mentha-1,4-dien-7-ol	18.476	0.60
25	Cis-2,6-Dimethyl-2,6-octadiene	19.981	0.97
26	Neryl acetate	20.511	0.40
27	Geranyl acetate	21.672	9.63
28	Germacrene-D	24.941	0.24
29	δ-Cadinene	26.602	0.24
30	1,7,7-terimethylbicyclo[2,2,1]hept-2-yl 3-methyl-2-butenoate	28.029	6.46
31	Spathulenol	28.324	0.36
32	Carotol	28.869	0.24
33	Alpha-Cadinol	30.753	0.29
34	Bisabolone oxide	31.619	2.37
35	Bisabolone oxide A	33.331	1.12
Total			98.64

**Figure 1:**
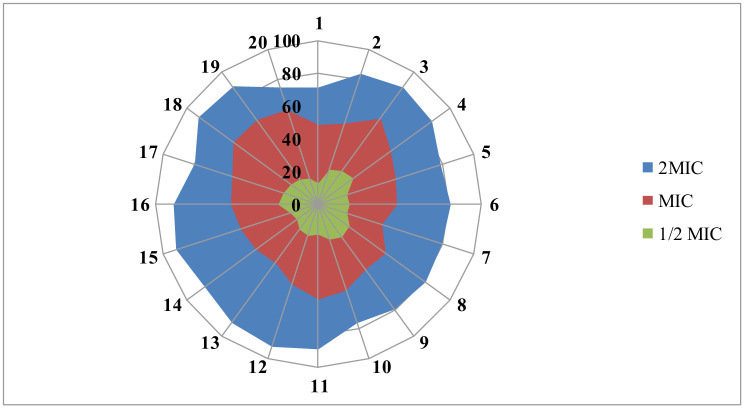
Comparison of the relative fungal inhibition (%) of *Bunium persicum* essence (2 MIC, MIC, and 1/2 MIC) against *Fusarium verticillioides* isolates. 1-20: number of studied isolates. MIC: minimum inhibitory concentration

The MICs and MFCs of *B. persicum* oil against *F. verticillioides* isolates are summarized in Table 2. The MIC values ranged from 2000 to 3000 µg/ml with a geometric mean value of 2556.8 μg/ml while the MFC values ranged from 3000 to 4000 µg/ml with a geometric mean value of 3591.6 μg/ml. The MFC/MIC ratio of *B. persicum* oil was 1.40, indicating the potent fungicidal activity of this oil against *F. verticillioides* isolates. The results showed that in all isolates, the relative fungal inhibition increased significantly with the increase of essence concentrations (*P*<0.05). As shown in Figure 1, the relative fungal inhibition in the presence of 2 MIC essence was calculated at 80-90% in most isolates.

**Table 2:** *In vitro* susceptibilities of *Fusarium verticillioides* isolates to *Bunium persicum* essence

Isolates	MIC	MFC	MFC/MIC Ratio
	µg/ml		
1	2500	3250	1.30
2	2750	4000	1.45
3	2750	4000	1.45
4	2750	3500	1.27
5	2500	3500	1.40
6	2000	3000	1.50
7	2500	3500	1.40
8	2500	4000	1.60
9	2000	3250	1.63
10	2500	3500	1.40
11	2500	4000	1.60
12	2750	4000	1.45
13	2750	3500	1.27
14	3000	4000	1.33
15	2000	3000	1.50
16	3000	4000	1.33
17	2750	4000	1.45
18	2500	3250	1.30
19	3000	4000	1.33
20	2500	3000	1.20

MIC: minimum inhibitory concentration

MFC: minimum fungicidal concentration

Based on the literature review, this study was the first investigation on the inhibitory effects of *B. persicum* essence on phytopathogenic *F. verticillioides* isolates. Several studies have been carried out about the antifungal efficacy of *B. persicum* against other phytopathogenic fungi. Sekine et al. [ 9 ] showed that *B. persicum* oil was responsible for its antifungal efficacy against phytopathogenic fungi, such as *Fusarium oxysporum*, *Verticillium dahliae*, *Botrytis cinerea*, and *Alternaria mali*. Khaledi and Hassani [ 15 ] in their study observed different values of MIC for treatments against the growth of *Colletotrichum lindemuthianum*. The MIC values for *B. persicum* oil ranged from 1010 to 2539 ppm. These various findings may be associated

with the use of different strains and testing methods; however, there are differences in essence origin and chemical components that play a pharmaceutical role.

**Figure 2:**
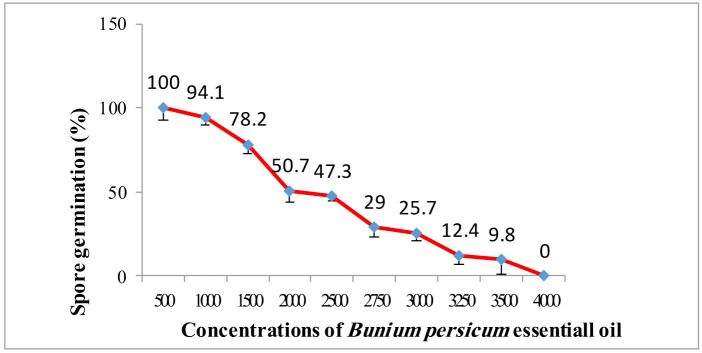
Effects of different concentrations of *Bunium persicum* essence on spore germination of *Fusarium verticillioides* isolates.

In this study, along with assessing the antifungal effect of *B. persicum* essence, its effect on spore germination inhibition was also evaluated. As shown in Figure 2, the efficacy of essence on spore germination was based on a dose-dependent pattern; accordingly, the spore germination can be reduced by increasing the essential oil concentrations. The results demonstrated that spore germination completely stopped at the concentration of 4000 µg/ml, while at concentrations less than 2000 µg/ml, more than 50% of the spores were germinated. Khalid and Hassani [ 15 ] found that *B. persicum* oil was significantly effective on

sporulation of C. lindemuthianum at 1× MIC concentration.

**Figure 3:**
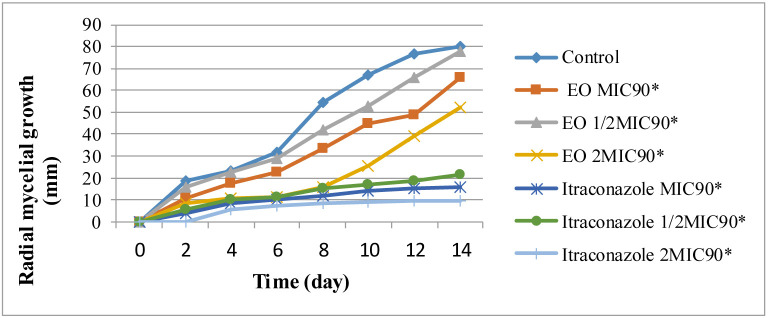
Effects of various concentrations of *Bunium persicum* oil and itraconazole (MIC_90_, 1/2 MIC_90_, and 2 MIC_90_) on radial mycelial growth of *Fusarium verticillioides* isolates.^*^Significant difference in comparison with control on day 7 (*P*<0.05). MIC: minimum inhibitory concentration

To our knowledge, no information exists about the efficacy of *B. persicum* on the radial mycelial growth of *F. verticillioides*. In the present investigation, the oil had the best inhibitory activity on the mycelia growth 

of *F. verticillioides* with a mean MIC value of less than 3591.6 μg/ml in vitro (Figure 3). Statistical analysis showed that *B. persicum* at MIC, 1/2 MIC, and 2 MIC significantly inhibited the radial mycelial growth of *F. verticillioides* strains after 7 days of incubation (*P*<0.05). This is parallel to the findings obtained by Sekine et al. [ 9 ] that revealed the best antifungal efficacy of *B. persicum* among 52 plant species tested. Behtoei et al. [ 20 ] revealed that *B. persicum* had a reducing effect on the mycelial growth of *F. oxysporum* isolates within the range of 42.45-100%. An investigation of the *B. persicum* sample indicated the very high inhibitory activity (59.61-100%) of the oil on radial mycelial growth of other phytopathogenic fungi, in particular *Rhizoctonia solani* and *Macrophomina phaseolina* [ 21 ].

In this study, since the MIC/MFC ratios were less than four in all studied isolates, the essence can be considered fungicidal. Results of the investigation of fungistatic and/or fungicidal activity by Khaledi et al. [ 21 ] indicated that the essence of *B. persicum* had fungicidal properties against various fungi at 1500 concentration. In this regard, Sharafati Chaleshtori et al. [ 16 ] demonstrated the changes in cell membrane structure upon exposure to *B. persicum* essence. Hydrophobic essence diffuses into *Fusarium* spp. resulting in cellular leakage of some reducing sugars, K^+^, Ca^2+^, Na^+^, and small molecules including proteins and nucleic acids which finally result in cell death. 

**Figure 4:**
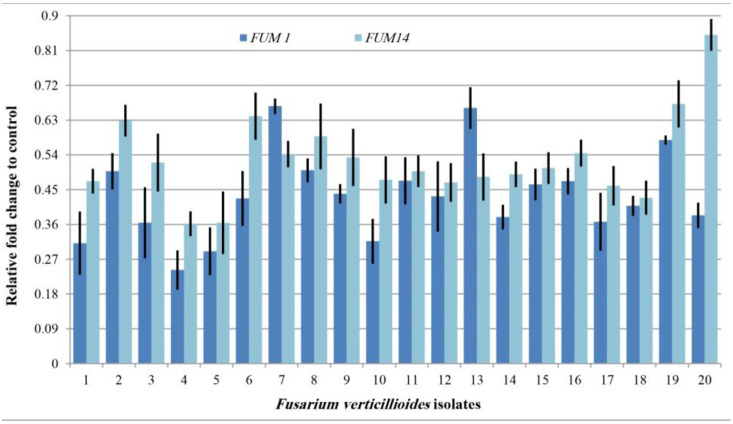
Effect of *Bunium persicum* essence on transcript levels of *FUM1* and *FUM14* genes of *Fusarium verticillioides* isolates. Error bars indicated the standard error of three independent replicates. Data calculated using the ^∆∆^CT method were expressed as relative units.

The used RT-PCR technique was a highly sensitive and specific method for the detection of the expressions of *FUM1* and *FUM14* genes in *F. verticillioides* isolates. The *FUM* cluster of *F. verticillioides* encodes biosynthetic enzymes as well as regulatory and transport proteins required for fumonisin production [ 22 ]. The *FUM1* encodes a polyketide synthase that is responsible for the synthesis of the fumonisin backbone. The *FUM14* gene is required for tricarballylic acid esterification and is also transcriptionally regulated [ 23 ]. The expression analyses of these two main genes, *FUM1* and *FUM14*, are shown in Figure 4. 

The genes expression of *FUM1* and *FUM14* were significantly reduced (*P*<0.05) after treatment with the essence in comparison with the genes in the control sample. The observed fold changes in target genes, *FUM1* and *FUM14*, were within the ranges of 0.24-0.66 fold (mean: 0.43 fold) and 0.36-0.85 fold (mean: 0.53 fold), respectively, at 2000 μg/ml concentration of *B. persicum* oil in comparison with the untreated control group (Table 3). Despite the known antifungal activity of *B. persicum*, no information has been reported about its effect on fumonisin biosynthesis at the gene expression level.

**Table 3:** Comparison of expressions of *FUM1* and *FUM14* genes in *Fusarium verticillioides* strains (F_1_-F_20_) before and after treatment with *Bunium persicum* essence.

Strain	FUM1 gene		FUM14 gene	
	ΔΔCT= Expression	2^ΔCT= Expression (RFC)*	ΔΔCT= Expression	2^ΔCT= Expression (RFC)
**F_1_	1.68	0.31	1.08	0.47
F_2_	1.00	0.50	0.67	0.63
F_3_	1.46	0.36	0.94	0.52
F_4_	2.05	0.24	1.47	0.36
F_5_	1.78	0.29	1.46	0.36
F_6_	1.22	0.43	0.64	0.64
F_7_	0.59	0.66	0.88	0.54
F_8_	1.00	0.50	0.77	0.59
F_9_	1.08	0.47	0.91	0.53
F_10_	1.66	0.32	1.07	0.47
F_11_	1.08	0.47	1.01	0.50
F_12_	1.21	0.43	1.09	0.47
F_13_	0.60	0.66	1.05	0.48
F_14_	1.40	0.38	1.03	0.49
F_15_	1.11	0.46	0.98	0.51
F_16_	1.08	0.47	0.88	0.54
F_17_	1.45	0.37	1.12	0.46
F_18_	1.29	0.41	1.22	0.43
F_19_	0.79	0.58	0.57	0.67
F_20_	1.38	0.38	0.23	0.85

* RFC: relative fold change

** F: *Fusarium verticillioides*

Overall, the results of the present study are in accordance with those of the investigations carried out by Lazzaro et al. [ 24 ] and Lozano-Ojalvo et al. [ 25 ], who showed the regulation of mycotoxin biosynthesis at a genetic level. Therefore, our findings suggested that the reduction of expressions of *FUM1* and *FUM14* genes might lead to a decrease in fumonisin level by *B. persicum* treatment. Lopez-Errasquin et al. [ 26 ] also found a good correspondence between the level of *FUM1* transcript and the production of fumonisin by *F. verticillioides* using a specific RT-PCR. In a previous study performed by Khosravi et al. [ 27 ], the *Cuminum cyminum* essence completely stopped the gene expression of *FUM1* in *F. verticillioides* in dose-dependent stimulation of antifungal concentrations. Furthermore, Divband et al. [ 6 ] in their study showed the downregulation of Tri4 gene expression from 4.04 to 6.27 fold in *F. verticillioides* isolates exposed to *Thymus vulgaris* essence.

## Conclusion

Based on these results, the essential oil of *B. persicum* had fungicidal and/or fungistatic activity against *F. verticillioides*. A lower concentration (2000 μg/ml ) of this oil was capable of reducing the expression of *FUM1* and *FUM14* as the main toxigenic genes of this fungus. Although the *in vitro* effect of natural antifungals does not always provide a good criterion for *in vivo* studies, additional investigations are necessary to prove their efficacy in field conditions as feed treatment and their possible phytotoxicity on plant/seed material.

## Acknowledgement

All authors would like to thank the Research Council of the University of Tehran, Tehran, Iran for the financial support of the research. 

## Authors’ contribution

A. K. designed the study. A. B. collected the data. A. K., H. S., and A. S. analyzed the data. A. S. and A. S. prepared the manuscript. All authors read and approved the final manuscript.

## Conflict of Interest

The authors declare no conflict of interest.

## Financial disclosure

The authors declare no financial disclosure.

## References

[1] Chen Y, Huang TT, Chen CJ, Hou YP, Zhang AF, Wang WX, et al ( 2012). Sensitivity of Fusarium verticillioides isolates from rice to a novel cyanoacrylate fungicide. Crop Prot.

[2] Thippeswamy S, Mohana DC, Abhishek RU, Manjunath K ( 2003). Effect of plant extracts on inhibition of Fusarium verticillioides growth and its toxin fumonisin B1 production. J Agric Sci Technol.

[3] Palacios SA, Susca A, Haidukowski M, Stea G, Cendoya E, Ramírez ML, et al ( 2015). Genetic variability and fumonisin production by Fusarium proliferatum isolated from durum wheat grains in Argentina. Int J Food Microbiol.

[4] Guo Z, Doll K, Dastjerdi R, Karlovsky P, Dehne HW, Altincicek B ( 2014). Effect of fungal colonization of wheat grains with Fusarium spp. on food choice weight gain and mortality of meal beetle larvae (Tenebrio molitor). PLoS One.

[5] Panda P, Aiko V, Mehta A ( 2015). Effect of aqueous extracts of Mentha arvensis (mint) and Piper betle (betel) on growth and citrinin production from toxigenic Penicillium citrinum. J Food Sci Technol.

[6] Divband K, Shokri H, Khosravi AR ( 2017). Down-regulatory effect of Thymus vulgaris L. on growth and Tri4 gene expression in Fusarium oxysporum strains. Microb Pathog.

[7] Azizi M, Davareenejad G, Bos R, Woerdenbag HJ, Kayser O (2009). Essential oil content and constituents of Black Zira (Bunium persicum [Boiss.] B. Fedtsch.) from Iran during field cultivation (Domestication). J Essen Oil Res.

[8] Oroojalian F, Kasra-Kermanshahi R, Azizi M, Bassami MR ( 2010). Phytochemical composition of the essential oils from three Apiaceae species and their antibacterial effects on food-borne pathogens. Food Chem.

[9] Sekine T, Sugano M, Majid A, Fujii Y ( 2007). Antifungal effects of volatile compounds from Black Zira (Bunium persicum) and other spices and herbs. J Chem Ecol.

[10] Council of Europe (1997). Methods of pharmacognosy. European pharmacopoeia.

[11] Clinical and Laboratory Standards Institute (CLSI) (2008). Reference method for broth dilution antifungal susceptibility testing of filamentous fungi; approved standard.

[12] Mohammadi A, Hashemi M, Hosseini SM ( 2015). Comparison of antifungal activities of various essential oils on the Phytophthora drechsleri, the causal agent of fruit decay. Iran J Microbiol.

[13] Kalagatur NK, Kamasani JR, Mudili V, Krishna K, Chauhan OP, Sreepathi MH ( 2018). Effect of high pressure processing on growth and mycotoxin production of Fusarium graminearum in maize. Food Biosci.

[14] Rocha LO, Barroso VM, Andrade LJ, Pereira GHA, Ferreira-Castro FL, Duarte AP, et al ( 2016). FUM gene expression profile and fumonisin production by Fusarium verticillioides inoculated in Bt and non-Bt maize. Front Microbiol.

[15] Khaledi N, Hassani F ( 2018). Antifungal activity of Bunium persicum essential oil and its constituents on growth and pathogenesis of Colletotrichum lindemuthianum. J Plant Protect Res.

[16] Sharafati Chaleshtori  F, Saholi M, Sharafati Chaleshtori  R ( 2018). Chemical composition, antioxidant and antibacterial activity of Bunium persicum, Eucalyptus globulus, and Rose Water on multidrug-resistant Listeria species. J Evid Based Integ Med.

[17] Shahsavari N, Barzegar M, Sahari MA, Naghdibadi H ( 2008). Antioxidant activity and chemical characterization of essential oil of Bunium persicum. Plant Foods Human Nutr.

[18] Azimzadeh M, Amiri R, Assareh MH, Bihamta MR, Forootan M ( 2012). Genetic diversity of Iranian Bunium persicum germplasm by morphological markers and essential oil components. J Med Plant Res.

[19] Naeini A, Ziglari T, Shokri H, Khosravi AR ( 2010). Assessment of growth-inhibiting effect of some plant essential oils on different Fusarium isolates. J Mycol Med.

[20] Behtoei H, Amini J, Javadi T, Sadeghi A ( 2012). Composition and in vitro antifungal activity of Bunium persicum, Carum copticum and Cinnamomum zeylanicum essential oils. J Med Plants Res.

[21] Khaledi N, Taheri P, Arighi S ( 2014). Antifungal activity of various essential oils against Rhizoctonia solani and Macrophomina phaseolina as major bean pathogens. J Appl Microbiol.

[22] Proctor RH, Van Hove F, Susca A, Stea G, Busman M, Vander Lee  T, et al ( 2013). Birth, death and horizontal gene transfer of the fumonisin byosinthetic gene cluster during the evolutionary diversification of Fusarium. Mol Microbiol.

[23] Fanelli F, Schmidt-Heydt M, Haidukowski M, Geisen R, Logrieco A, Mulè G ( 2012). Influence of light on growth, fumonisin biosynthesis and FUM1 gene expression by Fusarium proliferatum. Int J Food Microbiol.

[24] Lazzaro I, Susca A, Mule G, Ritieni A, Ferracane R, Marocco A ( 2012). Effects of temperature and water activity on FUM2 and FUM21 gene expression and fumonisin B production in Fusarium verticillioides. Eur J Plant Pathol.

[25] Lozano-Ojalvo D, Rodríguez A, Bernáldez V, Córdoba JJ, Rodríguez M ( 2013). Influence of temperature and substrate conditions on the omt-1 gene expression of Aspergillus parasiticus in relation to its aflatoxin production. Int J Food Microbiol.

[26] Lopez-Errasquın E, Vazquez C, Jimenez M, Gonzalez-Jaen MT ( 2007). Real-time RT-PCR assay to quantify the expression of FUM1 and FUM19 genes from the fumonisin-producing Fusarium verticillioides. J Microbiol Methods.

[27] Khosravi AR, Shokri H, Mokhtari AR ( 2015). Efficacy of Cuminum cyminum essential oil on FUM1 gene expression of fumonisin-producing Fusarium verticillioides strains. Avicenna J Phytomed.

